# Establishment of a canine urothelial carcinoma‐derived organoid biobank: A platform for comparative and translational research

**DOI:** 10.1002/ctm2.70645

**Published:** 2026-03-26

**Authors:** Christopher Zdyrski, Aleksandra Pawlak, Hannah F. Nicholson, Megan P. Corbett, Michael Catucci, John Cheville, Haejin Cho, Bryan J. Melvin, Jiayi Peng, Corey Saba, Hayden Hamsher, Steven G. Friedenberg, Andrew P. Woodward, Eugene Douglass, Jonathan P. Mochel, Karin Allenspach

**Affiliations:** ^1^ SMART Pharmacology Department of Biomedical Sciences Iowa State University Ames Iowa USA; ^2^ SMART Pharmacology Precision One Health Initiative University of Georgia Athens Georgia USA; ^3^ 3D Health Solutions Inc. Ames Iowa USA; ^4^ Department of Physiology and Pharmacology University of Georgia Athens Georgia USA; ^5^ Department of Pharmacology and Toxicology Faculty of Veterinary Medicine Wroclaw University of Environmental and Life Sciences Wroclaw Poland; ^6^ Department of Pathology College of Veterinary Medicine University of Georgia Athens Georgia USA; ^7^ Department of Laboratory Medicine and Pathology Mayo Clinic Rochester Minnesota USA; ^8^ Pharmaceutical and Biomedical Sciences Institute of Bioinformatics University of Georgia Athens Georgia USA; ^9^ Veterinary Teaching Hospital University of Georgia Athens Georgia USA; ^10^ Department of Veterinary Clinical Sciences University of Minnesota Saint Paul Minnesota USA

1

Dear Editor:

In this study, we describe the largest known organoid biobank derived from canine urothelial carcinoma patients, including phenotypic and molecular characterization. These organoids can be thawed, expanded, and screened for response to therapeutics, thereby expanding translational medicine approaches. Notably, canine urothelial carcinoma (UC) closely resembles human muscle‐invasive bladder cancer (MIBC) in terms of histopathology, molecular features, biological behavior, metastatic patterns, therapeutic response, and clinical outcome,^1^ underscoring the value of the dog as a comparative model.

A total of 27 organoid lines from 14 dogs were successfully established (Table S1); three (P1, P2, and P3) were previously described by our group with minimal characterization.^2^ From nine patients, only urine‐derived organoids were established. From two patients (P6 and P7), in addition to urine, we established tissue‐derived organoid lines, with P7 being sampled from two sites. From three patients (P12, P13, and P14), only tissue‐derived organoids were created, with two sample sites for P14. From P6, longitudinal sampling over approximately 7 months was performed, resulting in nine urine‐derived and two tissue‐derived organoid lines (Figure S1).

Organoids typically developed within 5 days. Urine‐derived organoids grew in high density over the first week, while tissue‐derived organoids grew and budded off the anchoring tissue fragments until being released via passaging. Both urine‐ and tissue‐derived cultures could be propagated for extended periods (>1 year), as tested on multiple organoid lines.

Once the cultures were expanded, no notable differences between tissue‐ or urine‐derived organoids were observed regarding their morphology and growth rate (Figure 1A–C). Slight variations between patients were observed, with some resembling more cystic structures (i.e., P4 and P8) and others budding structures (i.e., P2 and P3). Formalin‐fixed, paraffin‐embedded organoids had three main morphologies: solid, porous, or with a central lumen (Figure 1A–C).

**FIGURE 1:**
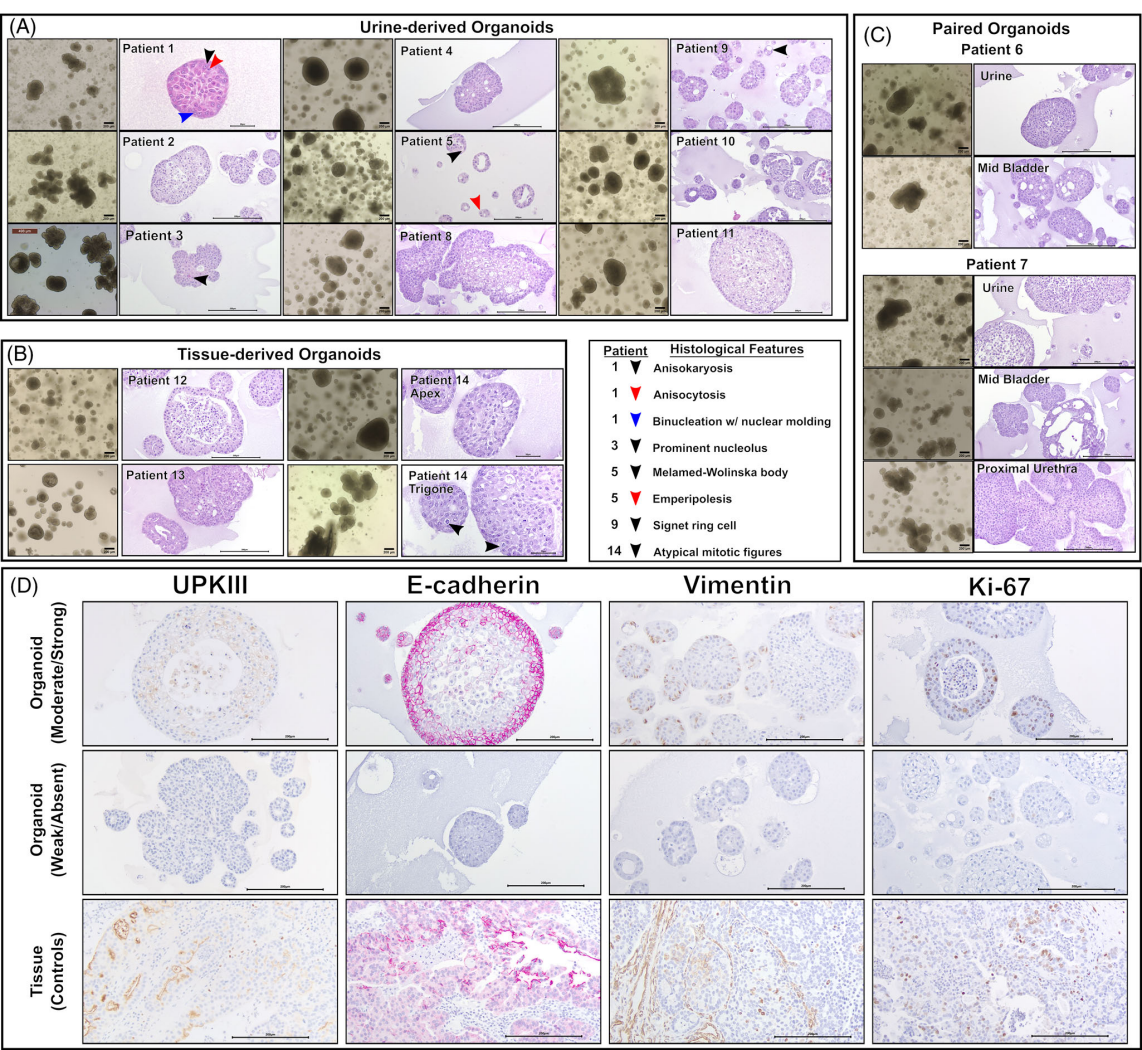
Examples of the morphological and histological phenotypes that can be found in (A) urine‐derived, (B) tissue‐derived, and (C) matched urine‐derived and tissue‐derived canine organoids from the same patients. Notable and representative histological features are denoted with arrows; arrow meanings are denoted in the key. Immunohistochemical appearance of canine UC‐derived organoids and corresponding tissues. (D) Immunohistochemistry (IHC) markers included UPKIII, E‐cadherin, Vimentin, and Ki‐67. Representative patients were used to demonstrate the variability of moderate/strong and weak/absent labeling across organoids and tissues. Scale bars are in micrometers.

Characterization of cell types was performed with immunohistochemistry when possible. Umbrella cells were identified using uroplakin III (UPKIII), with most organoid lines being immunonegative or faintly immunopositive. Additional markers included E‐cadherin (epithelial), Vimentin (mesenchymal‐like cells), and Ki‐67 (proliferative). Marker expression levels varied among patient‐derived organoid lines, likely reflecting underlying biological heterogeneity, patient‐to‐patient variability, time since passage, and/or total passage number (Figure 1D).

RNA sequencing (RNA‐seq) was used to assess the heterogeneity between different patient‐derived organoid lines and tissue samples. Principal component analysis showed that one cluster of all organoid lines separated from two distinct clusters from the tissue samples, even when comparing paired tissue/organoid samples from the same dog (Figure 2A). This was expected due to the absence of non‐epithelial cell types (immune, endothelial, etc.) in the organoids. Future studies could explore co‐culture of components of the tumor microenvironment with epithelial organoids.^3^ Tissue clusters were characterized by enrichment of genes associated with either luminal or basal molecular subtypes, as previously described.^4^, ^5^

**FIGURE 2:**
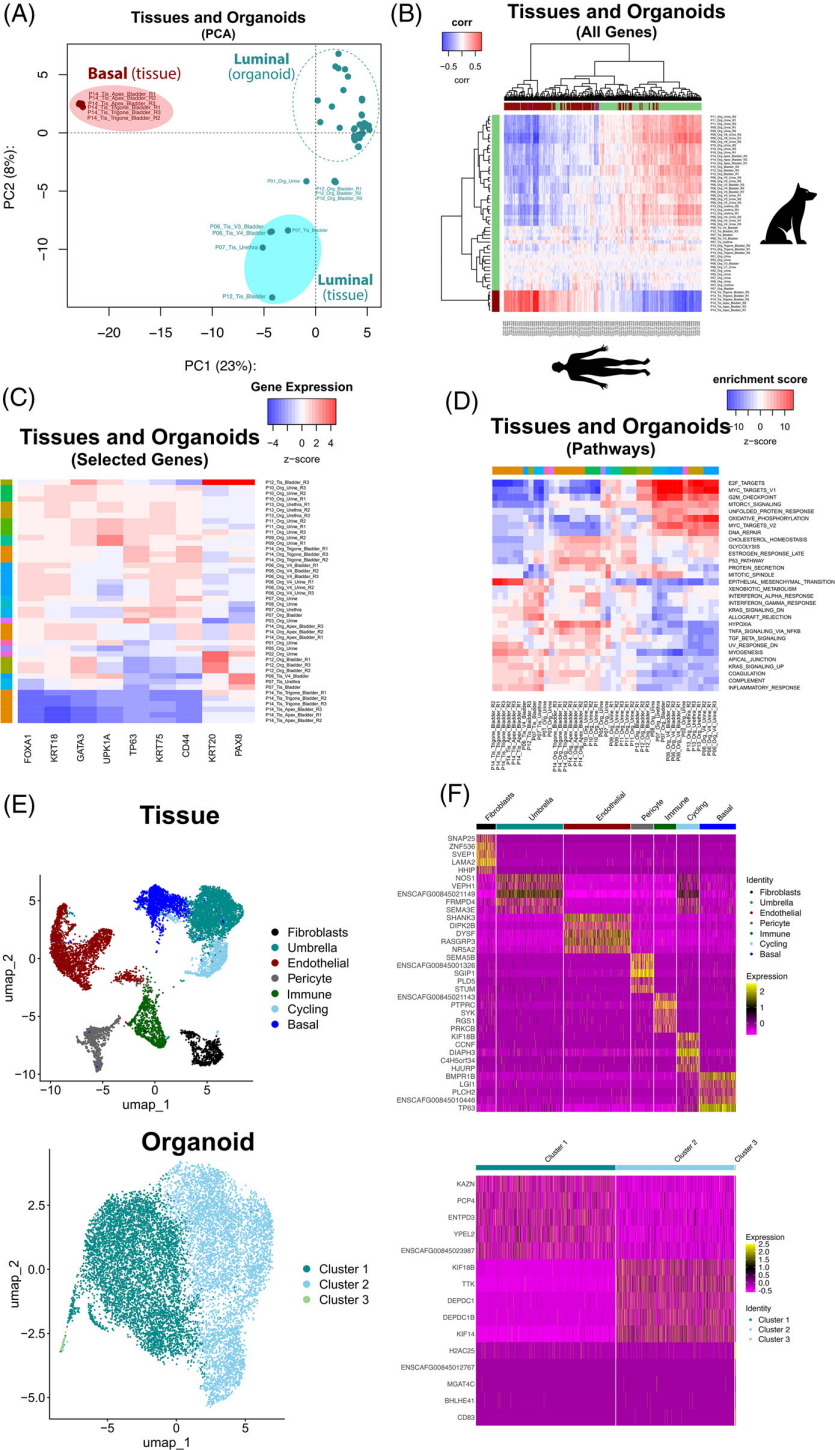
(A) Principal component analysis (PCA) showing the distribution of transcriptomes of organoid lines and tissues within the context of luminal (green) or basal (red) phenotypes according to The Cancer Genome Atlas (TCGA) classification.^4^ (B) A heatmap of all gene expression patterns across canine organoids and tissue samples compared to transcriptomic data publicly available from human MIBC tissue samples.^5^ (C) A heatmap displaying the expression profiles of selected genes that are relevant in UC across sample types. (D) Pathway analysis for hallmark gene sets across all samples.^10^ (E) Identification of cell clusters using snRNA‐seq for a matched canine UC‐derived organoid line and its parent tissue sample (P12). Annotated Uniform Manifold Approximation and Projection (UMAP) plots displaying distinct cell clusters (colored) identified in a paired canine tissue and organoid sample. (F) Heatmaps identifying the top markers for each cluster (upregulated = yellow and downregulated = purple, with respect to each other), expressed as average log2fold change.

Further media refinement may enable the transcriptomic signature of organoids to be shifted if needed,^5^ making them useful for cross‐species modeling. A comparison of transcriptomes from all tissue‐ and urine‐derived organoids is shown in a volcano plot (Figure S2). After false discovery rate (FDR) correction, 93.4% of genes showed no statistically significant difference between samples, with 6.6% meeting the *Q* < .05 threshold. This modest proportion is close to the rate expected under the null hypothesis, indicating broad transcriptomic similarity between the two sources. Several genes were upregulated in urine‐derived organoid lines compared to tissue‐derived, including *CXCL8* and *SLPI* (Figure S2b). For one patient (P12), we performed single‐nuclei RNA sequencing (snRNA‐seq) for both the tissue (seven clusters) and organoid (three clusters) culture (Figure 2).

We performed whole genome sequencing (WGS) to compare somatic mutations of paired tumor tissue and organoids. In general, our limited dataset comparing genes containing high‐, moderate‐ or low‐impact variants demonstrated that the mutated genes were similar between organoids and the corresponding tissues (Figure 3A). When analyzing specific variants of high‐ and moderate‐impact, however, most were found to be exclusive to either the tissue or the organoids (Figure 3B). This is likely due to differences in cellular composition, specifically, variation in cancer stem cell clone content, between samples. In addition, clonal selection imposed by in vitro conditions (i.e., media) is possible. In the three donors where matched tissue and organoids were available, the top 25 genes by variant count were tracked along with their predicted biological impact (moderate or high), with a subset from each patient being retained in the organoids (Figure 3C). Overall, these results demonstrate the importance of appropriately using organoid models when developing targeted therapies. Future studies highlighting conserved mutations for cross‐species therapeutic testing can enhance their translational value.

**FIGURE 3:**
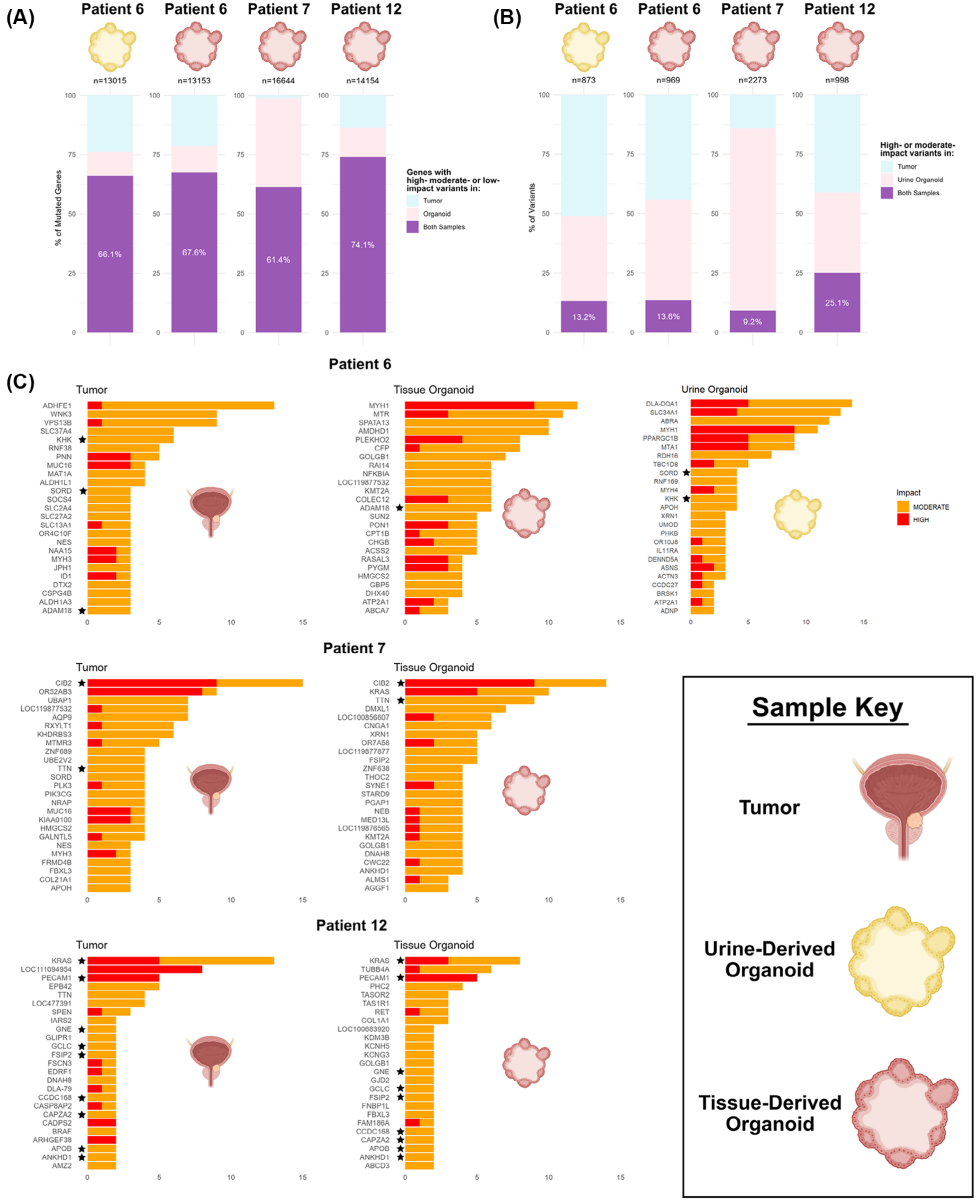
(A) Percentage of all genes containing high‐, moderate‐ or low‐impact variants in both samples (purple), unique to the organoid (pink), or unique to the tumor of origin (blue). (B) Percentage of identical high‐ or moderate‐impact variants in both samples (purple), unique to the organoid (pink), or unique to the tumor of origin (blue). (C) Top 25 genes by variant count with moderate‐impact (orange) or high‐impact (red) variants found in each sample. Stars denote the same gene present in both tumor and organoid. Organoid symbols represent urine‐derived organoids (yellow) and tissue‐derived organoids (pink). Created using BioRender.com.

To demonstrate the utility of UC‐derived organoids for functional drug sensitivity studies, we performed a proof‐of‐concept cytotoxicity study using vinblastine on two matched urine‐ and tissue‐derived organoids (P6 and P7) (Figure 4). Vinblastine displayed high potency across all patient‐derived organoids with low IC_50_ estimates (Table S2). It is important to note that methodological differences such as sample source, media composition, plate coating, extracellular matrix, culture duration, and protocols can impact cross‐study comparisons.^6^, ^7^, ^8^, ^9^ Nonetheless, this study lays the foundation to use this biobank for screening of novel treatment avenues with translational value.

**FIGURE 4:**
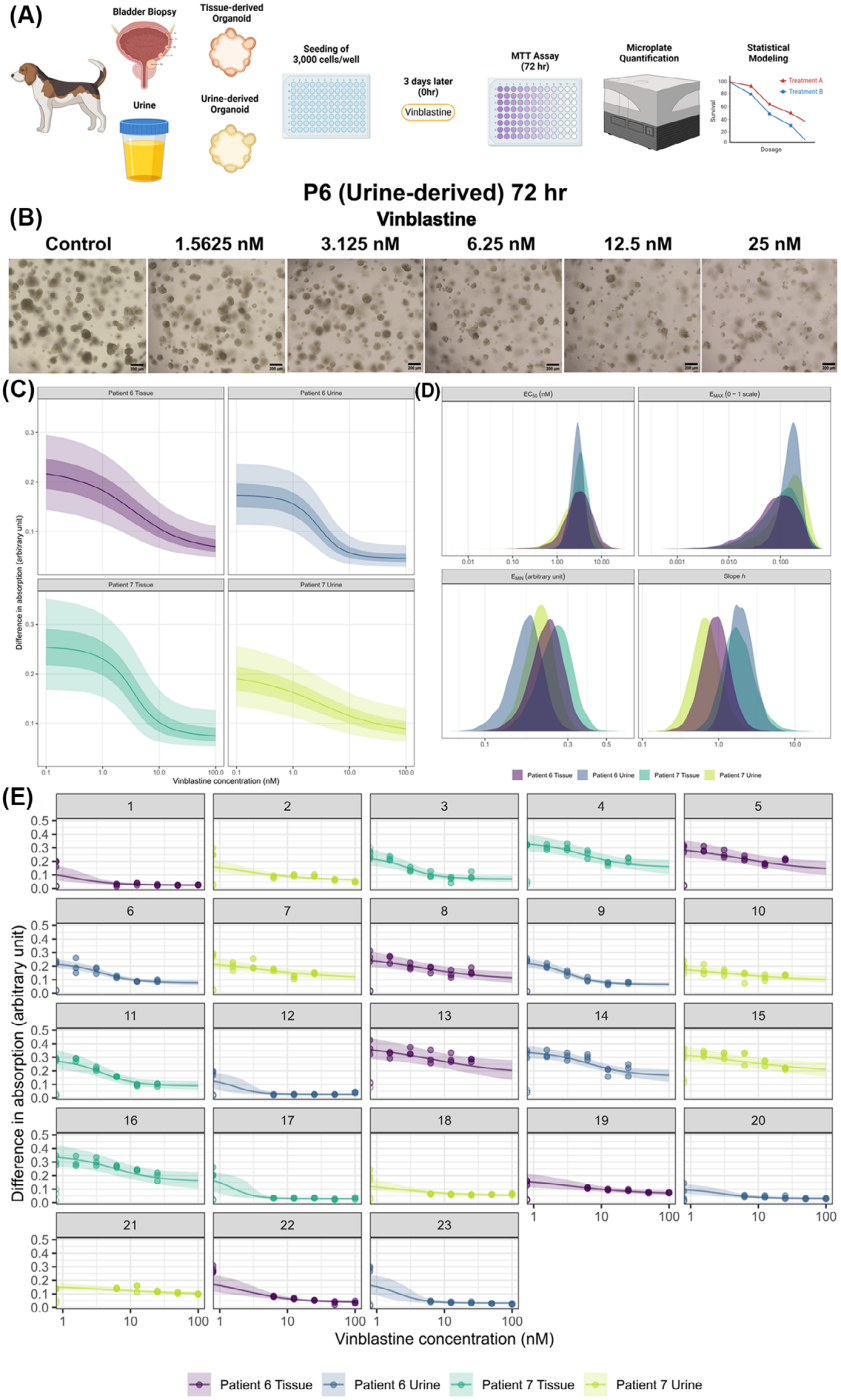
(A) Experimental workflow for treatment of urine‐ and tissue‐derived organoids, drug screening assay, and statistical modelling. (B) Brightfield images highlighting the morphological changes of controls and vinblastine‐treated organoids (controls: no treatment, five concentrations of vinblastine: 1.5625, 3.125, 6.25, 12.5 and 25 nM). Scale bars are in micrometers. (C) Posterior‐predicted dose‒response relationship for organoid viability (using Thiazolyl Blue Tetrazolium Bromide (MTT assay)) by vinblastine concentration (nM), by individual, for a hypothetical average plate (ignoring between‐plate variation). The solid line represents the posterior median predicted response, and the shaded fields indicate 50% and 90% credible regions demonstrating uncertainty in the dose‒response relationships. (D) Posterior probability distributions of the individual pharmacodynamic parameters. (E) Posterior‐predicted dose‒response relationships by plate. Points represent the observed data. Open points are the observations of dimethyl sulfoxide (DMSO)‐exposed controls, which are taken as the maximum possible inhibitory effect. The solid line represents the posterior median predicted response, and the field the 90% credible region. Panels indicate different plates, and colours indicate different subjects (urine and tissue specimens from two dogs). Created using BioRender.com.

This study is subject to several limitations. In the future, tumorigenicity should also be evaluated (i.e., xenografts) to confirm malignancy, as the presence of healthy organoids is possible. For urine‐derived samples, the noninvasive nature of sampling precluded access to matched tumor tissue and histopathology in most cases. Paired germline DNA samples were not analyzed, limiting our ability to distinguish somatic from germline variants. Finally, bulk RNA‐seq, while informative, masks cellular heterogeneity and may obscure the presence of minor populations. While snRNA‐seq overcomes some of these challenges, it was limited to one patient. Finally, many of these analyses were proof‐of‐concept and not carried out on the entire biobank, making the overall findings less generalizable.

In conclusion, we expanded our canine organoid biobank and report a comprehensive histological, molecular, and genomic characterization to investigate their potential and limitations. With limited human bladder cancer organoid biobanks available, and even fewer from canines, this biobank offers a valuable preclinical platform for translational research.

## AUTHOR CONTRIBUTIONS

Christopher Zdyrski, Aleksandra Pawlak, Hannah F. Nicholson, Megan P. Corbett, Michael Catucci, Bryan J. Melvin and Jiayi Peng collected data and expanded organoid lines. Eugene Douglass, Andrew P. Woodward, Haejin Cho, Hayden Hamsher and Steven G. Friedenberg completed data analysis and software writing. Megan P. Corbett and John Cheville completed histological review. Corey Saba assisted in sample collection. Christopher Zdyrski, Aleksandra Pawlak, Karin Allenspach and Jonathan P. Mochel wrote and edited the manuscript. Karin Allenspach and Jonathan P. Mochel funded the project. All the authors reviewed the manuscript.

## CONFLICT OF INTEREST STATEMENT

K. Allenspach is a co‐founder of LifEngine Animal Health and 3D Health Solutions. She serves as a consultant for Ceva Animal Health, Bioiberica, LifeDiagnostics, Antech Diagnostics, Purina, Hills, Boehringer Ingelheim and Mars. J.P. Mochel is a co‐founder of LifEngine Animal Health and 3D Health Solutions. Dr. Mochel is a consultant for Ceva Animal Health, Ethos Animal Health, LifEngine Animal Health, Dechra Ltd. and Boehringer Ingelheim. C. Zdyrski is the Director of Research and Product Development at 3D Health Solutions. The remaining authors declare they have no conflicts of interest.

## ETHICS STATEMENT

All sample collections derived from canine patients at Iowa State University (IACUC‐21‐250) and the University of Georgia (A2023 10‐002‐A1) were used under approved IACUC protocols. All biospecimen collections obtained from Purdue University were conducted under an approved IACUC protocol (#1111000124), and tissue or urine samples were shipped to ISU or UGA.

## Supporting information



Supporting Information

Supporting Information

Supporting Information

Supporting Information

## Data Availability

The stranded mRNA raw RNA‐seq reads and snRNA‐seq reads generated and analyzed in this study are available in the NCBI GEO (accession numbers GSE306810 and GSE306811). Additionally, three lines had their RNA‐seq files posted on the NIH ICDC portal (https://caninecommons.cancer.gov/#/study/ORGANOIDS01). The WGS raw files from this study are available in the Sequence Read Archive (NCBI‐SRA BioProject PRJNA1312855). RNA‐seq and cytotoxicity scripts are available on GitHub (https://github.com/chris‐zdyrski/TCC‐Organoids). Scripts used for post‐cram processing are also available on Github (https://github.com/Hexive‐UN‐03/cancer_code_release_2025). To process up to the cram stage, WAGS was used up until the cram rule (https://github.com/jonahcullen/wags).
