# Supplementary material for: Establishment of a canine urothelial carcinoma‐derived organoid biobank: A platform for comparative and translational research
Source: Clin Transl Med. 2026 Mar 26;16(4):e70645. doi: 10.1002/ctm2.70645 (PMC13140855; doi:10.1002/ctm2.70645)
Supplement: Supplementary file 4 — Supporting Information [file CTM2-16-e70645-s003.docx]

**Establishment of a Canine Urothelial Carcinoma-Derived Organoid Biobank: A Platform for Comparative and Translational Research**

**Christopher Zdyrski *^1,2,3^, Aleksandra Pawlak ^2,4,5^, Hannah F. Nicholson ^2^, Megan P. Corbett ^2,6^, Michael Catucci ^2^, John Cheville ^7^, Haejin Cho ^8^, Bryan J. Melvin ^2^, Jiayi Peng ^2^, Corey Saba ^9^, Hayden Hamsher ^10^, Steven G. Friedenberg ^10^, Andrew P. Woodward ^2^, Eugene Douglass ^8^, Jonathan P. Mochel ^1,2,3^, Karin Allenspach *^1,2,3^**

^1^ SMART Pharmacology, Department of Biomedical Sciences, Iowa State University, Ames, IA, USA

^2^ Present Address: SMART Pharmacology, Precision One Health Initiative, University of Georgia, Athens, GA, USA

^3^ 3D Health Solutions Inc., Ames, IA, USA

^4^ Department of Physiology and Pharmacology, University of Georgia, Athens, GA, USA

^5^ Department of Pharmacology and Toxicology, Faculty of Veterinary Medicine, Wroclaw

University of Environmental and Life Sciences, Wroclaw, Poland

^6^ Department of Pathology, College of Veterinary Medicine, University of Georgia, Athens, GA 30602, USA

^7^ Department of Laboratory Medicine and Pathology, Mayo Clinic, Rochester, MN, 55905

^8^ Pharmaceutical and Biomedical Sciences, Institute of Bioinformatics, University of Georgia, Athens, GA, USA

^9^ Veterinary Teaching Hospital, University of Georgia, Athens, GA, USA

^10^ Department of Veterinary Clinical Sciences, University of Minnesota, Saint Paul, MN, 55108, USA

*** Corresponding authors:**

Christopher Zdyrski (czdyrski@uga.edu)

Karin Allenspach (Karin.Allenspach@uga.edu)

**METHODS**

*Sample processing*

Samples for organoid culture were obtained from either urine or tissue biopsies of canine patients diagnosed with bladder cancer and, if collected at a different site, were placed in shipping media previously described (Sato et al., 2023), with the current composition reported more recently (Nicholson et al., 2026), immediately after sample collection. All sample collections derived from canine patients at Iowa State University (IACUC-21-250) and the University of Georgia (A2023 10-002-A1) were used under approved IACUC protocols. All biospecimen collections obtained from Purdue University were conducted under an approved IACUC protocol (#1111000124), and tissue or urine samples were shipped to ISU or UGA. Tissue samples were sectioned, with a subset immediately preserved in RNAlater (Invitrogen; AM7021), while the remaining pieces were submerged in shipping media for transport. Urine samples were collected by either catheter or free catch and spun at 600 x g for 3 minutes at 4°C. The supernatant was discarded, and ~10 mL of shipping media was added to the pellet and shipped to the lab on ice packs.

Upon arrival, urine samples were washed in PBS (Corning; 21-040-CM), and if red blood cells (RBC) were present, RBC lysis (Roche; 11814389001) was performed. After a final washing step, Matrigel (originally Corning; 356231 and later Corning; 356255) was added to the pellet, resuspended, and plated (30 µL per well) in a 24-well culture plate (Corning; 3524). The sample was incubated at 37°C for 20 minutes before the addition of culture media, which was slightly modified from the first patients to the final composition listed in Nicholson et al. (Nicholson et al., 2026). Tissue samples were minced with a scalpel, washed in Advanced DMEM/F12 (Gibco; 12634010), and then processed similarly as urine samples.

*Organoid culture*

Formed organoids were maintained as described elsewhere in detail (Nicholson et al., 2026). Organoids were cleaned or passed until a culture of the desired purity and cell number was obtained. Briefly, organoids were resuspended in Cell Recovery Solution (Corning; 354270), incubated at 4°C for 10 minutes, and spun (100 x g for 5 minutes at 4°C). If passing, organoids were then incubated with TryPLE Express (Gibco; 12604-021) for 10 minutes and washed in DMEM/F12 (100 x g for 5 minutes at 4°C) prior to resuspension in fresh Matrigel, plating, and addition of fresh culture media. The expanded organoids were then frozen using CryoStor CS10 (Biolife Solutions; 210102) and stored in liquid nitrogen.

*Fixation and H&E staining*

For hematoxylin and eosin (H&E) staining, organoid media was removed, and 500 µL of Formalin-acetic acid-alcohol, (FAA, composition in Gabriel et al. 2022) made from ethanol (VWR: 71002-512), acetic acid (Fisher; A38500), formaldehyde (Fisher; F79P4), and MilliQ water, was added to each well (Gabriel et al., 2022). After 24 hours, FAA was replaced with 70% ethanol, which was made from diluting 100% ethanol, and samples were paraffin-embedded, mounted on slides, and stained at the Iowa State University or the University of Georgia Histopathology laboratory. Tissues were fixed in formalin and paraffin-embedded according to standard histology procedures.

*Immunohistochemistry*

Slides were cut and labeled at the University of Georgia Histopathology core for Vimentin V9 (BioGenex; MU074-UC), E-cadherin (BD Biosciences; 610181), and Ki-67 (Cell Marque; 275R-16). Slides were additionally sent to Cornell University for UPKIII labeling (Fitzgerald Industries; 10R-U103ax). Slides were then imaged using an Olympus BX41 microscope with a BioVid 4k camera (LW Scientific). Images were color-corrected, and scale bars were added with ToupView, version x64 131 4.11.23945.20231121 (LW Scientific).

*RNA Extractions*

Organoids were preserved for bulk RNA-sequencing by removing them from Matrigel using Cell Recovery Solution, as previously described (Nicholson et al., 2026). The cell pellet was resuspended in 100 µL PBS, moved to a cryovial containing 900 µL RNAlater, and stored at -80°C. For RNA extractions, organoid samples were thawed and transferred to 15 mL tubes, washed with 2 mL of PBS, and centrifuged at 1,200 x g (4°C) for 5 minutes. Then the supernatant was removed, and the cell pellet was resuspended in 1 mL of Trizol (Invitrogen; 15596026). Next, each sample was briefly vortexed. Tissues that were either flash frozen or stored in RNAlater (if stored in RNAlater a wash with PBS was done) were transferred from cryovials to microcentrifuge tubes, 800 µL of Trizol was added to each tube, and tissues were homogenized using a pestle. Both samples (tissue and organoids) were left at room temperature for five minutes prior to centrifuging at 12,000 x g (4°C) for 10 minutes. Supernatant was then transferred to a microcentrifuge tube, and 160 µL or 200 µL of chloroform (Alfa Aesar; J67241) was added for tissues and organoids, respectively. The samples were then vigorously mixed by shaking for 20 seconds and after sitting at room temperature for 2-3 minutes, the samples were spun at 10,000 x g for 18 minutes (4°C). The aqueous top layer was collected and moved to a sterile RNase-free tube before adding an equal amount of 100% RNA-free ethanol (Fisher; BP2818-500). Up to 700 µL was loaded in a Qiagen RNeasy column and collection tube (RNeasy Mini kit; Qiagen; 74104). Samples were then centrifuged at 8,000 x g for 30 seconds, the collection tube waste was discarded, and DNase treatment was performed according to Qiagen’s protocol (Qiagen; 79254). In a new collection tube, 500 µL buffer RPE was added to the column, and the samples were again spun. After discarding the flow-through, this step was repeated, with samples now spun for 2 minutes at 8,000 x g. The flow-through was discarded again, and the samples were centrifuged at the same settings for 1 minute. Then, 50 µL of RNase-free water (Sigma; W4502-50ML) was added to the column and incubated for 2 minutes. Samples were centrifuged twice at 8,000 x g for 1 minute. RNA concentration was determined with a Nanodrop ND-1000 Spectrophotometer (Thermo Fisher Scientific), and samples were kept at -80°C until shipped.

*Bulk RNA sequencing*

Three of the samples, P1, P2, and P3, were prepped and sequenced at the Mayo Clinic using the names OR-B, OR-A, and OR-E, respectively. All other samples had library preparation and sequencing conducted at Azenta Life Sciences (South Plainfield, NJ, USA) as follows: RNA samples were quantified using Qubit 2.0 Fluorometer (ThermoFisher Scientific, Waltham, MA, USA), and RNA integrity was checked with 4200 TapeStation (Agilent Technologies, Palo Alto, CA, USA). Strand-specific RNA sequencing library was prepared by using NEBNext Ultra II Directional RNA Library Prep Kit for Illumina following manufacturer’s instructions (NEB, Ipswich, MA, USA). Briefly, the enriched RNAs were fragmented for 8 minutes at 94°C. First strand and second strand cDNA were subsequently synthesized. The second strand of cDNA was marked by incorporating dUTP during the synthesis. cDNA fragments were adenylated at 3’ ends, and indexed adapter was ligated to cDNA fragments. Limited cycle PCR was used for library enrichment. The incorporated dUTP in second strand cDNA quenched the amplification of second strand, which helped to preserve the strand specificity. The sequencing library was validated on the Agilent TapeStation (Agilent Technologies, Palo Alto, CA, USA) and quantified by using Qubit 2.0 Fluorometer (ThermoFisher Scientific, Waltham, MA, USA), as well as by quantitative PCR (KAPA Biosystems, Wilmington, MA, USA). The sequencing libraries were multiplexed and clustered onto a flow cell on the Illumina NovaSeq instrument according to manufacturer’s instructions. The samples were sequenced using a 2x150bp Paired End (PE) configuration. Image analysis and base calling were conducted by the NovaSeq Control Software (NCS). Raw sequence data (.bcl files) generated from Illumina NovaSeq was converted into fastq files and de-multiplexed using Illumina bcl2fastq 2.20 software. One mis-match was allowed for index sequence identification.

*Nuclei isolation and cell counting*

For single-nuclei samples, frozen samples were received at Azenta, South Plainfield, NJ, USA in dry-ice and stored in liquid nitrogen until further processing. Nuclei extraction was performed using the Miltenyi Nuclei Extraction Buffer (Miltenyi Biotec, Auburn, CA, USA) following manufacturer’s guidelines with gentle MACS Dissociation and C tubes. Upon isolation, the nuclei were counted using AO/PI dye on the Nexcelom Cellaca MX before loading onto the Chromium Controller.

*3’ RNA library preparation and sequencing*

Single-nuclei RNA libraries were generated using the Chromium Single Cell 3’ kit (10X Genomics, CA, USA). Loading onto the Chromium Controller was performed to target capture ~6,000 GEMs per sample for downstream analysis and processed through the Chromium Controller following the standard manufacturer’s specifications. The sequencing libraries were evaluated for quality on the Agilent TapeStation (Agilent Technologies, Palo Alto, CA, USA) and quantified using Qubit 2.0 Fluorometer (Invitrogen, Carlsbad, CA). Libraries were quantified using qPCR (Applied Biosystems, Carlsbad, CA, USA) prior to loading onto an Illumina NovaSeq XPlus instrument. The samples were sequenced at a configuration compatible with the recommended guidelines as outlined by 10X Genomics. Raw sequence data (.bcl files) were converted into fastq files and de-multiplexed using the 10X Genomics’ cellranger mkfastq command.

*Bulk and single-nuclei RNA sequencing analysis*

After sequencing, read quality was assessed using FastQC, and high-quality reads were mapped to the canine reference genome CanFam3.1. Kallisto or CellRanger was used to conduct read alignment and gene expression counts for bulk and single-nuclei RNA sequencing, respectively (Bray et al., 2016; Zheng et al., 2017). Next, single-nuclei RNA sequencing data was normalized and log transformed using R package Seurat (Butler et al., 2018; Hao et al., 2021, 2024; Satija et al., 2015; Stuart et al., 2019) R and corresponding R scripts were then used for data preparation procedures, analysis procedures, and visualization (R Core Team, 2018). The data was stored on GitHub. Gene Set Enrichment Analysis with the fgsea package was used for pathway analysis, and gene signatures were acquired from the Molecular Signatures Database (MSigDB) (Korotkevich et al.; Liberzon et al., 2011).

*DNA extractions*

Organoids were preserved for whole genome sequencing (WGS) by cleaning as before, washing in PBS, and transferring to a cryovial for a final spin. Dry cell pellet or tissue samples were stored at -80°C. A Qiagen Blood and Tissue Kit was used on both tissues and organoid cultures to extract DNA. The manufacturer’s protocol was followed with the addition of RNase A (Thermo Fisher Ref: EN0531) to degrade excess RNA. A Nanodrop was used to quantify the DNA, and samples were immediately stored at -80°C. Samples were then shipped on dry ice to Genewiz for QC, preparation, and sequencing.

*Whole genome sequencing*

Genomic DNA was quantified using the Qubit 2.0 Fluorometer (ThermoFisher Scientific, Waltham, MA, USA). NEBNext® Ultra™ DNA Library Prep Kit for Illumina, clustering, and sequencing reagents were used throughout the process following the manufacturer’s recommendations. Briefly, the genomic DNA was fragmented by acoustic shearing with a Covaris S220 instrument. Fragmented DNA was cleaned up and end-repaired. Adapters were ligated after adenylation of the 3’ ends followed by enrichment by limited cycle PCR. DNA libraries were validated using a High Sensitivity D1000 ScreenTape on the Agilent TapeStation (Agilent Technologies, Palo Alto, CA, USA) and were quantified using Qubit 2.0 Fluorometer. The DNA libraries were also quantified by real time PCR (Applied Biosystems, Carlsbad, CA, USA). The sequencing libraries were multiplexed and clustered onto a flow cell on the Illumina NovaSeq instrument according to manufacturer’s instructions. The samples were sequenced using a 2x150bp Paired End (PE) configuration. Image analysis and base calling were conducted by the NovaSeq Control Software (NCS). Raw sequence data (.bcl files) generated from Illumina NovaSeq was converted into fastq files and de-multiplexed using Illumina bcl2fastq 2.20 software. One mismatch was allowed for index sequence identification.

*Whole genome analysis*

FASTQ files obtained from sequencing were aligned to the UU_Cfam_GSD_1.0 canine reference genome (Wang et al., 2021) using the Whole Animal Genome Sequencing (WAGS) pipeline (Cullen and Friedenberg, 2023) to generate binary alignment maps (BAM files). Each sample’s BAM file was individually genotyped using Mutect2 (Benjamin et al., 2019) in tumor-only mode with a --max-mnp-distance parameter set to zero. The resulting genotype records were merged into a single multi-sample file and normalized using bcftools (Danecek et al., 2021). To remove suspected germline variants, the combined file was filtered with GATK SelectVariants --discordance (Auwera and O’Connor, 2020), excluding all variants present in an internal database of whole genome sequences derived from 3,023 dogs, wolves, and coyotes of 402 diverse breeds. This database includes 1,971 dogs, wolves, and coyotes released by the Dog10K consortium (Meadows et al., 2023). High-confidence variant sites were retained by selecting variants where at least one sample had sequencing depth (DP) > 5, variant allele frequency (AF) > 0.01, and tumor log-odds score (TLOD) > 6.

*Cytotoxicity study*

Vinblastine (sulfate) (Cayman Chemical; 11762) was purchased as powder, dissolved in DMSO (Millipore Sigma; D2650-100ML) to prepare a stock solution, and stored at −20°C until use. Organoids derived from two patients (P6 and P7) from both urine and tissue samples were used in the study. The cells were plated at a density of 3,000 cells per well in 10 µL of Matrigel in 96-well plates (Corning; 3596) 3 days before the experiment and then were exposed to increasing concentrations of drug for 72 hours (DMSO concentration never exceeded 0.1%, which is considered harmless to the cells). Cells were plated in 3 replicates (3 wells), and the entire experiment was repeated 3 times. Vinblastine was tested in a concentration range of 1.5 – 100 nM. Cells incubated in the medium alone served as a control, 4% DMSO was used as a death control. After 72 hours, 10 μL of MTT solution (5 mg/mL) (Sigma Aldrich; M5655) was added to each well, as MTT assays have been previously used in organoid studies (Bode et al., 2019). After 2 hours, medium with MTT was removed and 50 µL of 2% SDS was added to dissolve Matrigel, for another hour. Then, 100 μL of DMSO was added, and after dissolution of the content, absorbance was measured at 570 nm with a spectrophotometric microplate reader (Spark, Tecan, Maennedorf, Switzerland).

## Pharmacodynamic data analysis

Analysis of the dose-response data was conducted in Stan (Carpenter et al., 2017) via R (R Core Team, 2024) with visualization from packages ‘ggplot2’ (Wickham, 2016) and ‘ggdist’ (Kay, 2024). The structural model was four-parameter log-logistic following (Ritz et al., 2015), allowing for between-subject and between-plate variation in each of the pharmacodynamic parameters $E_{\mathrm{MIN}}$, $E_{\mathrm{MAX}}$, ${EC}_{50}$, and $H$ (slope). Subjects were allowed to vary independently, and between-plate variation was presumed to be normal on the ${log}_{e}$-scale. The response variable was the difference in absorbance between 570 nm and the reference wavelength 630 nm. Its residual error was defined as lognormal.

To provide an absolute scale for the effect, the $E_{MAX}$ was defined on the logistic scale (constraining its value to $(0,1)$, exclusive), and transformed to the observed scale as a multiple of the difference between the estimated response for DMSO controls, which were taken to represent the minimum possible response, and the $E_{MIN}$. The $E_{MIN}$ was constrained to be larger than the response for the DMSO controls such that the form of the dose-response relationship was forced to be negative (inhibitory). This specification follows (Long et al., 2026).

The prior information for all pharmacodynamic parameters and their variabilities was intended to be weak, simply constraining them to physically realistic values. The prior specification is available in the study code. Principles for specification of prior models for veterinary pharmacology applications were described by the author (Woodward, 2024). Goodness-of-fit of the completed models was assessed visually by comparing posterior predictions to the observations at the subject and plate level (**Figure 4**).

**Supplemental Tables and Figures**

**Supplemental Table 1**. Patient metadata including patient unique identifier, sampling location, breed, sex, and age. Female spayed = FS, Female intact = FI, Male neutered = MN.

**Supplemental Table 2**. IC_50_ estimates and other parameters given for vinblastine across the two patients for both tissue- and urine-derived organoids.

**Supplemental Table 3.** Tecan Spark microplate reader settings used to assess cellular metabolic activity.


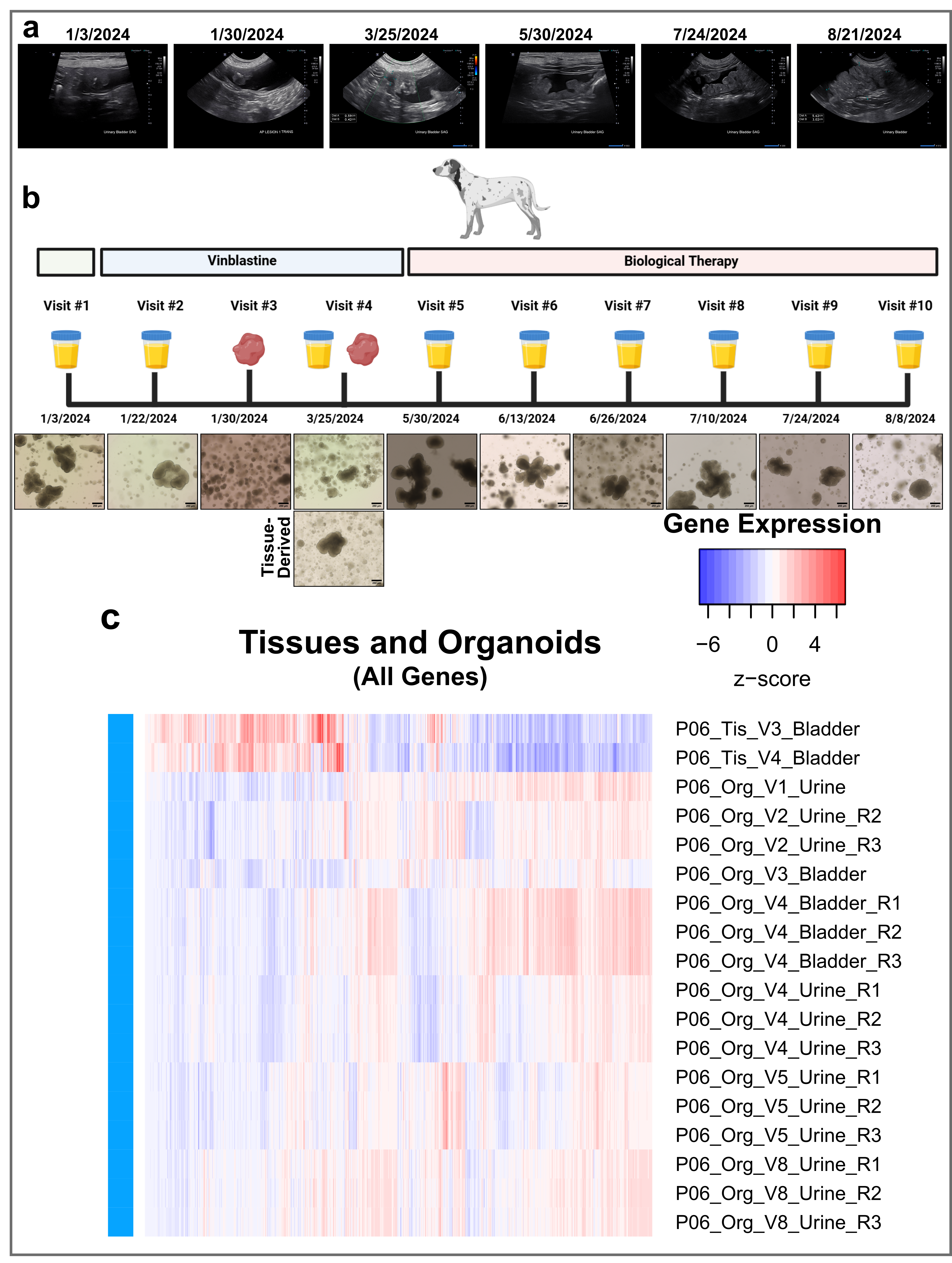


**Longitudinal sampling and generation of urothelial carcinoma-derived organoids from one canine patient.**

**Supplemental Figure 1:** (**a**) Ultrasound imaging of the *in vivo* patient from visits throughout the growth of the tumor, from the same patient with which organoids were successfully derived. (**b**) Types of samples collected over time and morphological photos for each one. Figure was made using Biorender.com. (**c**) RNA sequencing heatmap of all genes for longitudinal urine and tissue-derived organoid lines and tissue biopsies derived from P6.





**Supplemental Figure 2:** (**a**) PCA showing heterogeneity between transcriptomes of individual canine UC-derived organoid lines. Organoid lines derived from the same patients are depicted in the same color. (**b**) Volcano plot comparing the transcriptomes of all tissue- versus urine-derived organoids, where the dotted line represents significance. (**c**) snRNA-seq analysis showing expression of the top 5 genes per cluster with the dot size depicting the percentage of cells in a class and dot color corresponding to the average expression level across all cells within a class (red = higher expression, blue = lower expression). (**d**) Selected genes from each cluster plotted against all other clusters.

**REFERENCES**

Auwera, G. van der, and O’Connor, B.D. (2020). Genomics in the cloud : using Docker, GATK, and WDL in Terra (Sebastopol, CA: O’Reilly Media).

Benjamin, D., Sato, T., Cibulskis, K., Getz, G., Stewart, C., and Lichtenstein, L. (2019). Calling Somatic SNVs and Indels with Mutect2. https://doi.org/10.1101/861054.

Bode, K.J., Mueller, S., Schweinlin, M., Metzger, M., and Brunner, T. (2019). A fast and simple fluorometric method to detect cell death in 3D intestinal organoids. Biotechniques *67*, 23–28. https://doi.org/10.2144/btn-2019-0023.

Bray, N.L., Pimentel, H., Melsted, P., and Pachter, L. (2016). Near-optimal probabilistic RNA-seq quantification. Nat. Biotechnol. *34*, 525–527. https://doi.org/10.1038/nbt.3519.

Butler, A., Hoffman, P., Smibert, P., Papalexi, E., and Satija, R. (2018). Integrating single-cell transcriptomic data across different conditions, technologies, and species. Nat. Biotechnol. *36*, 411–420. https://doi.org/10.1038/nbt.4096.

Carpenter, B., Gelman, A., Hoffman, M.D., Lee, D., Goodrich, B., Betancourt, M., Brubaker, M.A., Guo, J., Li, P., and Riddell, A. (2017). Stan: A probabilistic programming language. J. Stat. Softw. *76*. https://doi.org/10.18637/jss.v076.i01.

Cullen, J.N., and Friedenberg, S.G. (2023). Whole Animal Genome Sequencing: user-friendly, rapid, containerized pipelines for processing, variant discovery, and annotation of short-read whole genome sequencing data. G3: Genes, Genomes, Genetics *13*. https://doi.org/10.1093/g3journal/jkad117.

Danecek, P., Bonfield, J.K., Liddle, J., Marshall, J., Ohan, V., Pollard, M.O., Whitwham, A., Keane, T., McCarthy, S.A., and Davies, R.M. (2021). Twelve years of SAMtools and BCFtools. Gigascience *10*. https://doi.org/10.1093/gigascience/giab008.

Gabriel, V., Zdyrski, C., Sahoo, D.K., Dao, K., Bourgois-Mochel, A., Kopper, J., Zeng, X.-L., Estes, M.K., Mochel, J.P., and Allenspach, K. (2022). Standardization and Maintenance of 3D Canine Hepatic and Intestinal Organoid Cultures for Use in Biomedical Research. Journal of Visualized Experiments https://doi.org/10.3791/63515.

Hao, Y., Hao, S., Andersen-Nissen, E., Mauck, W.M., Zheng, S., Butler, A., Lee, M.J., Wilk, A.J., Darby, C., Zager, M., et al. (2021). Integrated analysis of multimodal single-cell data. Cell *184*, 3573-3587.e29. https://doi.org/10.1016/j.cell.2021.04.048.

Hao, Y., Stuart, T., Kowalski, M.H., Choudhary, S., Hoffman, P., Hartman, A., Srivastava, A., Molla, G., Madad, S., Fernandez-Granda, C., et al. (2024). Dictionary learning for integrative, multimodal and scalable single-cell analysis. Nat. Biotechnol. *42*, 293–304. https://doi.org/10.1038/s41587-023-01767-y.

Kay, M. (2024). ggdist: Visualizations of Distributions and Uncertainty in the Grammar of Graphics. IEEE Trans. Vis. Comput. Graph. *30*, 414–424. https://doi.org/10.1109/TVCG.2023.3327195.

Korotkevich, G., Sukhov, V., Budin, N., Shpak, B., Artyomov, M.N., and Sergushichev, A. Fast gene set enrichment analysis. https://doi.org/10.1101/060012.

Liberzon, A., Subramanian, A., Pinchback, R., Thorvaldsdóttir, H., Tamayo, P., and Mesirov, J.P. (2011). Molecular signatures database (MSigDB) 3.0. Bioinformatics *27*, 1739–1740. https://doi.org/10.1093/bioinformatics/btr260.

Long, M., Peng, Y., Gade, S., Nicholson, H., Catucci, M., Zdyrski, C., Pawlak, A., Corbett, M., Saba, C., Laver, T., et al. (2026). Innovative In Vitro-In Silico Platform for Dose–Response Modeling in Canine Bladder Cancer: A 3D Organoid- and Mathematics-Based Approach. AAPS J. *28*, 74. https://doi.org/10.1208/s12248-026-01214-1.

Meadows, J.R.S., Kidd, J.M., Wang, G.D., Parker, H.G., Schall, P.Z., Bianchi, M., Christmas, M.J., Bougiouri, K., Buckley, R.M., Hitte, C., et al. (2023). Genome sequencing of 2000 canids by the Dog10K consortium advances the understanding of demography, genome function and architecture. Genome Biol. *24*. https://doi.org/10.1186/s13059-023-03023-7.

Nicholson, H.F., Zdyrski, C., Leyson, C.M., Corbett, M.P., Kumar, N., Catucci, M., Melvin, B.J., Stabler, L.J., Lakdawala, S.S., Douglass, E., et al. (2026). Development and characterization of chicken lung organoids for in vitro modeling of avian influenza virus-host cell interaction. Sci. Rep. *16*, 1572. https://doi.org/10.1038/s41598-025-30641-x.

R Core Team (2018). R: A language and environment for statistical computing .

R Core Team (2024). R: A Language and Environment for Statistical Computing .

Ritz, C., Baty, F., Streibig, J.C., and Gerhard, D. (2015). Dose-response analysis using R. PLoS One *10*. https://doi.org/10.1371/journal.pone.0146021.

Satija, R., Farrell, J.A., Gennert, D., Schier, A.F., and Regev, A. (2015). Spatial reconstruction of single-cell gene expression data. Nat. Biotechnol. *33*, 495–502. https://doi.org/10.1038/nbt.3192.

Sato, Y., Elbadawy, M., Suzuki, K., Tsunedomi, R., Nagano, H., Ishihara, Y., Yamamoto, H., Azakami, D., Uchide, T., and Nabeta, R. (2023). Establishment of an experimental model of canine malignant mesothelioma organoid culture using a three-dimensional culture method. Biomedicine & Pharmacotherapy *162*, 114651. .

Stuart, T., Butler, A., Hoffman, P., Hafemeister, C., Papalexi, E., Mauck, W.M., Hao, Y., Stoeckius, M., Smibert, P., and Satija, R. (2019). Comprehensive Integration of Single-Cell Data. Cell *177*, 1888-1902.e21. https://doi.org/10.1016/j.cell.2019.05.031.

Wang, C., Wallerman, O., Arendt, M.L., Sundström, E., Karlsson, Å., Nordin, J., Mäkeläinen, S., Pielberg, G.R., Hanson, J., Ohlsson, Å., et al. (2021). A novel canine reference genome resolves genomic architecture and uncovers transcript complexity. Commun. Biol. *4*. https://doi.org/10.1038/s42003-021-01698-x.

Wickham, H. (2016). ggplot2: Elegant Graphics for Data Analysis (Springer-Verlag).

Woodward, A.P. (2024). Bayesian estimation in veterinary pharmacology: A conceptual and practical introduction. J. Vet. Pharmacol. Ther. *47*, 322–352. https://doi.org/10.1111/jvp.13433.

Zheng, G.X.Y., Terry, J.M., Belgrader, P., Ryvkin, P., Bent, Z.W., Wilson, R., Ziraldo, S.B., Wheeler, T.D., McDermott, G.P., Zhu, J., et al. (2017). Massively parallel digital transcriptional profiling of single cells. Nat. Commun. *8*. https://doi.org/10.1038/ncomms14049.
